# N7-Methylguanosine-Related lncRNAs: Integrated Analysis Associated With Prognosis and Progression in Clear Cell Renal Cell Carcinoma

**DOI:** 10.3389/fgene.2022.871899

**Published:** 2022-04-13

**Authors:** Jie Ming, Chunyang Wang

**Affiliations:** Department of Urology, The First Affiliated Hospital of Harbin Medical University, Harbin, China

**Keywords:** clear cell renal cell carcinoma, N7-methylguanosine, lncRNA, prognosis, tumor microenvironment, tumor mutation burden

## Abstract

N7-Methylguanosine (m7G) and long non-coding RNAs (lncRNAs) have been widely reported to play an important role in cancer. However, there is little known about the relationship between m7G-related lncRNAs and clear cell renal cell carcinoma (ccRCC). To find new potential biomarkers and construct an m7G-related lncRNA prognostic signature for ccRCC, we retrieved transcriptome data and clinical data from The Cancer Genome Atlas (TCGA), and divided the entire set into train set and test set with the ratio of 1:1 randomly. The m7G-related lncRNAs were identified by Pearson correlation analysis (|coefficients| > 0.4, and *p* < 0.001). Then we performed the univariate Cox regression and least absolute shrinkage and selection operator (LASSO) Cox regression analysis to construct a 12 m7G-related lncRNA prognostic signature. Next, principal component analysis (PCA), the Kaplan–Meier method, time-dependent receiver operating characteristics (ROC) were made to verify and evaluate the risk signature. A nomogram based on the risk signature and clinical parameters was developed and showed high accuracy and reliability for predicting the overall survival (OS). Functional enrichment analysis (GO, KEGG and GSEA) was used to investigate the potential biological pathways. We also performed the analysis of tumor mutation burden (TMB), immunological analysis including immune scores, immune cell infiltration (ICI), immune function, tumor immune escape (TIE) and immunotherapeutic drug in our study. In conclusion, using the 12 m7G-related lncRNA risk signature as a prognostic indicator may offer us insight into the oncogenesis and treatment response prediction of ccRCC.

## Introduction

Renal cell carcinoma (RCC) is the seventh most commonly diagnosed cancer in the world, with an estimated 403,000 people diagnosed annually and 175,000 individuals dying of it in 2018 (Padala et al., 2020). RCC has also been the deadliest urological cancer with the 5-years survival rate of 12% in a dismal late stage (Padala et al., 2020). Clear cell renal cell carcinoma (ccRCC), as the most common type, accounts for approximately 75% of diagnoses of RCC, and its major causative agents are tobacco smoking, elevated blood pressure, obesity, occupational exposure (like trichloroethylene) and so on (Pischon et al., 2006; Moore et al., 2010; Colt et al., 2011; Tsivian et al., 2011). Surgery is an effective treatment for early-stage ccRCC, while there are approximately 30% of patients facing recurrence and distant metastases of tumors after surgery surgical operations. Metastatic ccRCC is insensitive to conventional radiotherapy and chemotherapy, and despite the development of tyrosine kinase inhibitors (TKIs) and immune checkpoint blockade (ICB) like Sorafenib, Nivolumab and Ipilimumab, there have been still some reports of tumor recurrence and progression (Ljungberg et al., 2019; Mori et al., 2021). Therefore, it is necessary for further exploration of the biological features, molecular mechanisms and immune system interactions of ccRCC.

N7-Methylguanosine (m7G), one of the most common forms of base modifications in post-transcriptional regulation, is the methylation of the guanosine base at the nitrogen-7 position. The methylated guanine is joint to the first transcribed nucleotide of messenger RNA (mRNA) through a 5’-5’- triphosphate linker, which is the formation of 7-methylguanosine mRNA cap (m7G mRNA cap) first reported by the author Wei and Furuichi in 1975 (Furuichi et al., 1975; Wei et al., 1975). Recently, it has shown that there were different versions of m7G cap in different organisms and in various RNA types. In eukaryotes, cap variants can be distinguished by the methylation pattern of the first few 5'-nucleotides of an m7G-capped RNA body, referred to as cap 0, cap 1, cap 2 structure, etc. Cap 0 is the structure mentioned above, which is the m7G cap without additional methylations in RNA and more common in lower eukaryotes (Furuichi, 2015). Cap 1 or cap 2 can be produced when cap 0 undergo further methylations at the 2'-O position within the ribose of the first or within the first and second nucleotide, both of which are more common in higher organisms like mammals (Furuichi, 2015; Warminski et al., 2017). In addition to mRNA, m7G has also been detected internally in different types of RNAs, such as rRNAs, tRNAs, miRNAs and miRNA precursors (Furuichi, 2015; Pandolfini et al., 2019).

m7G caps play an important role in coordinating the various biological processes that occur throughout the life cycle of various types of RNAs. In the nucleus, the m7G cap (cap 0) is involved in the pre-mRNA splicing (Pabis et al., 2013), transcription termination, exosomal degradation (Andersen et al., 2013) and nuclear export through recruiting and binding to the nuclear cap-binding complex (CBC) consisting of CBP80 and CBP20(Topisirovic et al., 2011). When exported into the cytoplasm, CBC remains bound to the cap, successively recruiting a series of eukaryotic initiation factors such as eIF4G, eIF4A, eIF3G, eIF4III and CBC-dependent translation initiation factor (CTIF) for the pioneer round of translation where there will be nonsense-mediated decay (Choe et al., 2012; Choe et al., 2014; Halstead et al., 2015). Afterwards, the combination of importin-β and importin-α results in the replacement of CBC by eIF4E which interacts with the eIF4F complex and initiates the steady-state rounds of translation (Maquat et al., 2010a; Maquat et al., 2010b). Besides, the poly (A) binding protein PABP1, bound to the poly (A) tail of mRNA, interacts with eIF4F complex to form a pseudo-circular structure, which ensures translation of full-length translating mRNA (Preiss and Hentze, 1998; Smith et al., 2014). Concerning 2'-O methylation of cellular RNA, recent studies revealed that it was helpful to distinguish self from non-self RNA through cellular sensors RIG-I and MDA5 and effectors IFIT1 and IFIT5 of the Type I interferon, such the distinction of viral from host RNA (Daffis et al., 2010; Devarkar et al., 2016; Ramanathan et al., 2016).

At present, a large number of genes have been found to participate in the regulation of m7G modification. Through binding to the m7G cap of the target mRNA, AGO2 was able to inhibit the recruitment of eIF4E, thus repressing the steady translation (Kiriakidou et al., 2007). It has also been reported that eIF4E could not only drive RNMT expression to promote the maturation of m7G cap but also bind directly to the cap to facilitate mRNA nuclear export and translation efficiency (Osborne et al., 2022). Kumar et al. observed that IFIT5 was capable of inhibiting the translation by recognizing the triphosphate bridge which linked the m7G cap to the first nucleotide at the 5' end of mRNA and then reducing the formation of the 48S translational initiation complex (Kumar et al., 2014). DCP2 was found to contain a highly conserved but functional 23-amino acid motif (GX_5_EX_7_REUXEEXGU) necessary for the decapping activity against m7G caps on intact mRNAs (Wang et al., 2002). METTL1 and WDR4 protein were the most well-characterized catalyzing enzymes that mediated m7G modification in tRNA at a ‘‘RAGGU’’ motif, which was required for self-renewal and differentiation of embryonic stem cells and the stability of tertiary N13-N22-m7G46 by improving the geometry of tRNA (Shi and Moore, 2000; Alexandrov et al., 2002; Lin et al., 2018), while WBSCR22 was responsible for the methylation of G1639 on 18s rRNA and the processing of 18S rRNA precursors (Haag et al., 2015).

Naturally, accumulating evidence revealed the importance of m7G-related genes in pathogenesis and progression of renal cancer. For example, the AC + AA genotypes of AGO2 rs*4961280* was significantly associated with renal caner progression, and the overexpression of AGO2 in the circulatory system has also been regarded as a biomarker of higher aggressiveness of renal tumors (Teixeira et al., 2021). It has been already found for the critical roles of eIF4E in tumor cell growth, proliferation and migration while inhibition of eIF4E would attenuate the malignancy of tumor cells and increase the sensitivity of RCC cells to chemotherapy and immunotherapy (Woodard et al., 2008; Cao et al., 2018). Lo et al. pointed that the formation of IFIT5-XRN1 complex contributed to the invasion of RCC cells by facilitating the degradation of tumor suppressor miRNA (miR-363and miR-128) and the expression of tumor-promoting genes Slug and ZEB1 (Lo et al., 2019). Besides, substantial evidence also revealed the connection between the aberrant m7G RNA modification and the development of a wide range of cancers such as prostate cancer, liver cancer, lung cancer, bladder cancer, etc. (D’Abronzo et al., 2017; Chen et al., 2021; Ma J et al., 2021; Ying et al., 2021)

Long non-coding RNAs (lncRNAs) are RNAs with a length exceeding 200 nucleotides, but do not encode proteins (Derrien et al., 2012). It has shown that lncRNAs impose an important effect on the proliferation and migration of tumor cells (Gonzalez et al., 2015; Melé et al., 2017; Ransohoff et al., 2018). However, few prognostic models of m7G-related lncRNAs have emerged. Therefore, we aimed to construct an m7G-related lncRNA prognostic signature to evaluate and improve prognosis in patients with ccRCC. On the basis of the signature, we further performed the analysis of tumor microenvironment (TME), ESTIMATE scores, immune cell infiltration (ICI), immune function, tumor immune escape (TIE), prediction of drug sensitivity and tumor mutation burden (TMB) by using the relevant public data.

## Materials and Methods

### Data Source

In order to acquire the synthetic data matrices about kidney renal clear cell carcinoma (KIRC-adenomas and adenocarcinomas) and normal kidney tissues, the datasets of HTSeq-FPKM and the relevant clinical information were downloaded from The Cancer Genome Atlas (TCGA) database (https://portal.gdc.cancer.gov/). And a total of 539 tumor samples and 72 normal samples were included in this study. To reduce bias in the statistical analysis, we excluded patients with a follow-up time of less than 30 days. We divided the patients into a train set and a test set using the ratio 1:1 randomly.

The dataset of tumor mutation was also downloaded from TCGA (simple nucleotide variation-Masked Somatic Mutation), and we chose the file “TCGA.KIRC.varscan.ee42d944-ccbf-4406-9e7c-ffe1a0a4a1d7.DR-10.0.somatic.maf” to conduct the further investigation of TMB (Supplementary Material 1). The analysis of ICI and immune functions were conducted by using CIBERSORT algorithm (Supplementary Material 2). ESTIMATE scores including stromal scores and immune scores were calculated through the “estimate” R package (Supplementary Material 3). The data of TIE were downloaded from TIDE (http://tide.dfci.harvard.edu/) (Supplementary Material 4).

### Selection of m7G-Related Genes and lncRNAs

Based on the method in the previous report (Li et al., 2021; Zhao et al., 2021), we used the keywords “7-Methylguanosine”, “N7-Methylguanosine” and “m7G” to find m7G-related genes from the database Genecards (https://www.genecards.org/), OMIM (https://omim.org/), NCBI (https://www.ncbi.nlm.nih.gov/) and Gene Set Enrichment Analysis (GSEA, http://www.gsea-msigdb.org/gsea/index.jsp). Finally, we got a total of 152 m7G-related genes after deleting duplicate items (Supplementary Material 5). The GSEA database contains three m7G-related gene sets: M18244, M26066 and M26714. Pearson correlation analysis (|coefficients| > 0.4, and *p* < 0.001) was performed between 152 m7G-related genes and all of lncRNAs in the combined matrices. And then we screened the m7G-related lncRNAs with the criteria of |log_2_ Fold Change (FC)| > 1, false discovery rate (FDR) < 0.05, and finally there were 2121 differentially expressed m7G-related lncRNAs.

### Establishment and Validation of the Risk Signature

Using the overall survival data in the clinical information from TCGA, we performed univariate Cox proportional hazard regression to screen 641 m7G-related lncRNAs with the standard of *p* < 0.05. Afterwards, the least absolute shrinkage and selection operator (LASSO) Cox regression was run with 10-fold cross-validation and a *p*-value of 0.05 along with 1,000 cycles. Each cycle was set up with a random stimulation 1,000 times as a measure of preventing overfitting. Then a model with 12 m7G-related lncRNAs was established. We calculated the risk score by multiplying the LASSO Cox coefficients [coef (lncRNA)] with the gene expression [exp (lncRNA)], which was the following formula:

$$\text{risk score}=\overset{n}{\underset{k=1}{\sum }}\text{coef}\left({\text{IncRNA}}^{\text{k}}\right)\text{*exp}\left({\text{IncRNA}}^{\text{k}}\right)$$

Then patients were divided into low-risk and high-risk group by taking the median risk score as the cut-off point.

### Independent Risk Factors For Overall Survival

We utilized uni-Cox and multi-Cox regression analysis to identify whether the risk score was the independent risk factors of overall survival (OS) in patients with ccRCC. Receiver operating characteristic (ROC) curves were used to validate the accuracy of the model.

### Nomogram and Calibration

With rms and survival R packages, we used the parameters of the risk score, age, gender, tumor grade, T stage and distant metastasis and tumor stage to create a nomogram for the 1-, 3-, and 5-years OS. A calibration curve based on the Hosmer–Lemeshow test was used to determine if the prediction outcome was in good agreement with the practical result.

### Functional Enrichment Analysis

We divided all of patients into two groups of high and low risk according to the above risk signature, and screened the differentially expressed m7G-related genes with the criteria of |log_2_ FC| > 1 and *p* < 0.05 between two groups. And then we conducted GO and KEGG enrichment analysis for differentially expressed genes. The GSEA software (Version. 4.1.0) was applied to analyze the pathways enriched in low-risk and high-risk group with curated gene set of c2.cp.kegg.v7.5.symbols.gmt. We screened the enrichment results with the criteria of nominal *p* value <5% and FDR <25%.

### Drug Sensitivity

Using the R package pRRophetic, we assessed the treatment response in patients of two groups as determined by the half-maximal inhibitory concentration (IC50) on the Genomics of Cancer Drug Sensitivity (GDSC) (https://www. cancerrxgene.org/).

### Statistical Analysis

Spearman coefficients were used to explore the correlations between m1A regulators. The differences between categorical variables were compared using Chi-square or Fisher exact tests. Continuous variables normally distributed between risk groups were compared through a student *t*-test. Alternatively, the Mann-Whitney U test was chosen. A univariate Cox regression analysis was used to calculate the hazard ratio (HR). Multivariate Cox regression analysis was used to identify the independent prognostic factors of the risk score. The receiver operating characteristic (ROC) curves and calibration curves were performed to evaluate the specificity and sensitivity of the prognostic model. Maftools R package was applied to visualize genetic changes in high-risk and low-risk groups according to mutation profiles. Strawberry Perl was utilized to synthesize the data matrix. All of the statistical analysis were conducted *via* R software 4.1.1. The significance threshold was *p* < 0.05.

## Results

### Information of Samples and Genes

**FIGURE 1:**
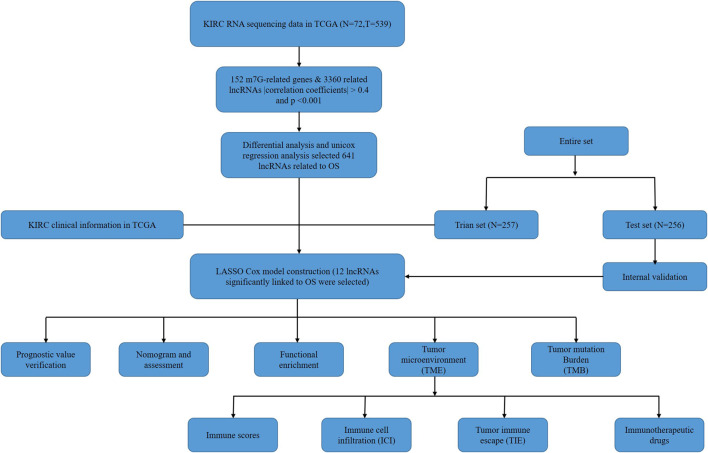
Flow chart of the study.

**FIGURE 2:**
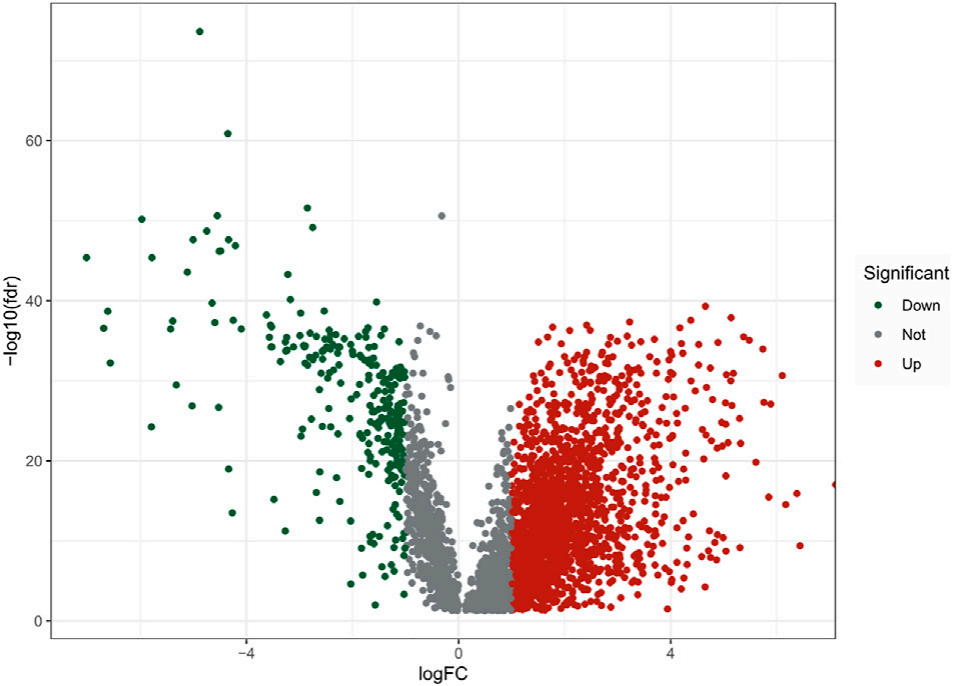
The volcano plot of 2121 differentially expressed m7G-realted lncRNAs.

The flow chart of this study was shown in Figure 1. There were a total of 611 samples including 539 tumor tissues and 72 normal ones from TCGA. According to the expression of 152 m7G-related genes and Pearson correlation analysis (|coefficients| > 0.4 and *p* < 0.001), we acquired 3,360 m7G-related lncRNAs. Then there were 2121 differentially expressed lncRNAs (|log_2_FC| > 1 and *p* < 0.05), among which 1850 lncRNAs were upregulated and 271 lncRNAs downregulated (Figure 2).

### Construction and Verification of the Risk Signature

**FIGURE 3:**
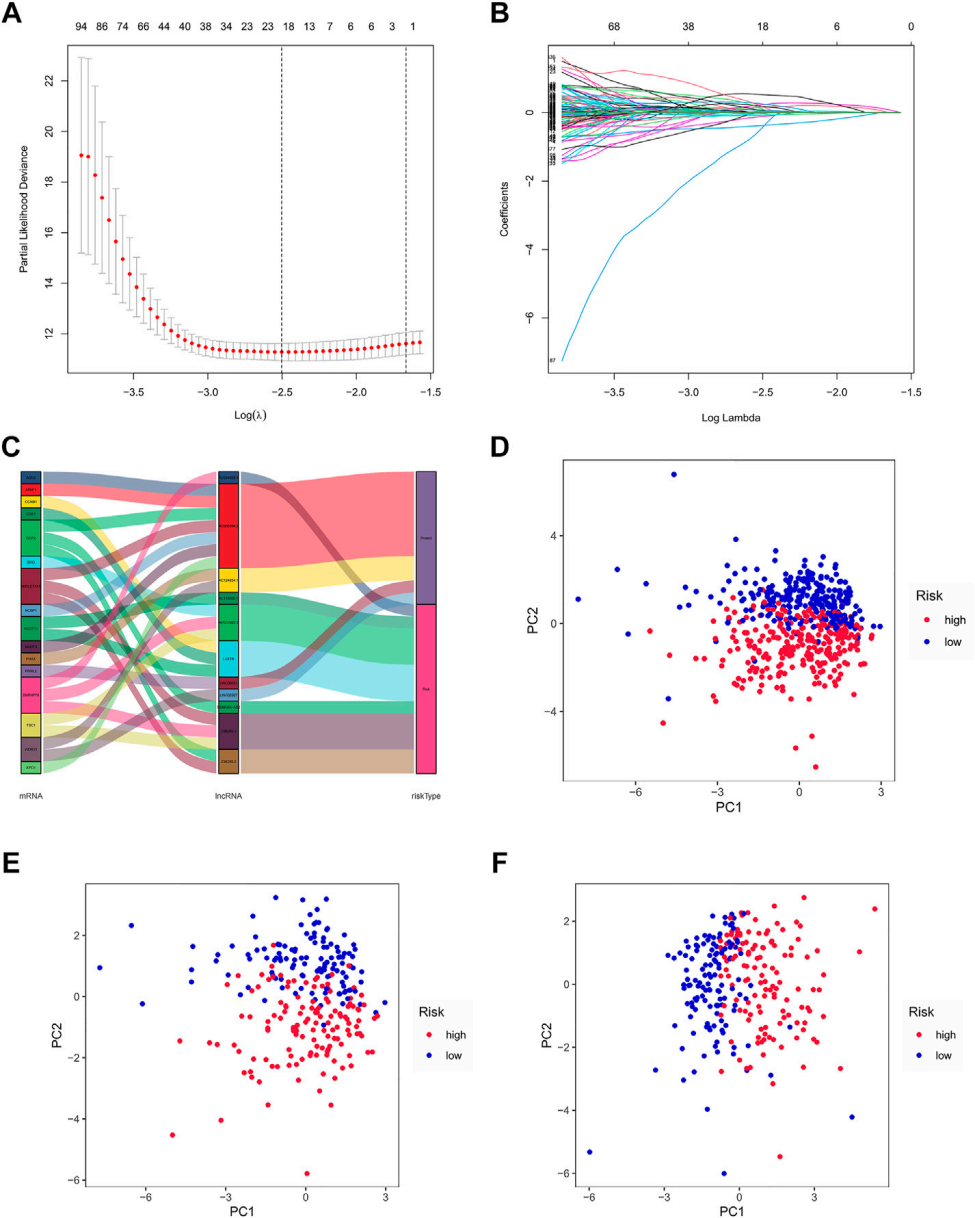
Exhibition of m7G-related lncRNAs prognostic signature in ccRCC. **(A)** The 10-fold cross-validation for variable selection in the LASSO model. **(B)** The LASSO coefficient profile of 12 m7G-related lncRNAs. **(C)** The Sankey diagram of m7G genes and related lncRNAs **(D–F)** The PCA of two risk groups in the entire set, test set and train set, respectively.

By univariate Cox regression analysis, we obtained 641 lncRNAs significantly associated with OS. To avoid overfitting prognostic signature, we further performed LASSO Cox regression (Figures 3A,B) and finally 12 lncRNAs were considerably associated with prognosis, of which 8 were risk genes and 4 were protective genes in the Sankey diagram (Figure 3C). And the risk scores were calculated by the following formula:

$$\begin{array}{l} \mathrm{Risk}\;\mathrm{score}=SEMA6A-AS2*\left(-0.5862\right)+Z98200.1*\left(1.1913\right)\\ +AC002070.1*\left(-0.4729\right)\\ +AC008264.2*\left(-7.992\right)+AC004908.1*\left(0.4678\right)\\ +AC124854.1*\left(-0.1546\right)+AL118508.1*\left(1.1958\right)\\ +LASTR*\left(0.6089\right)+LINC02027*\left(-0.3809\right)\\ +Z99289.2*\left(0.9331\right)+AP001893.3*\left(1.9744\right)\\ +LINC00551*\left(-2.2175\right) \end{array}$$

By taking the median risk score as the cut-off point, patients in the train set, test set and entire set were divided into high and low-risk group, respectively. The different PCA between two groups showed that our risk signature has good discrimination of the samples in our study (Figures 3D–F).

**FIGURE 4:**
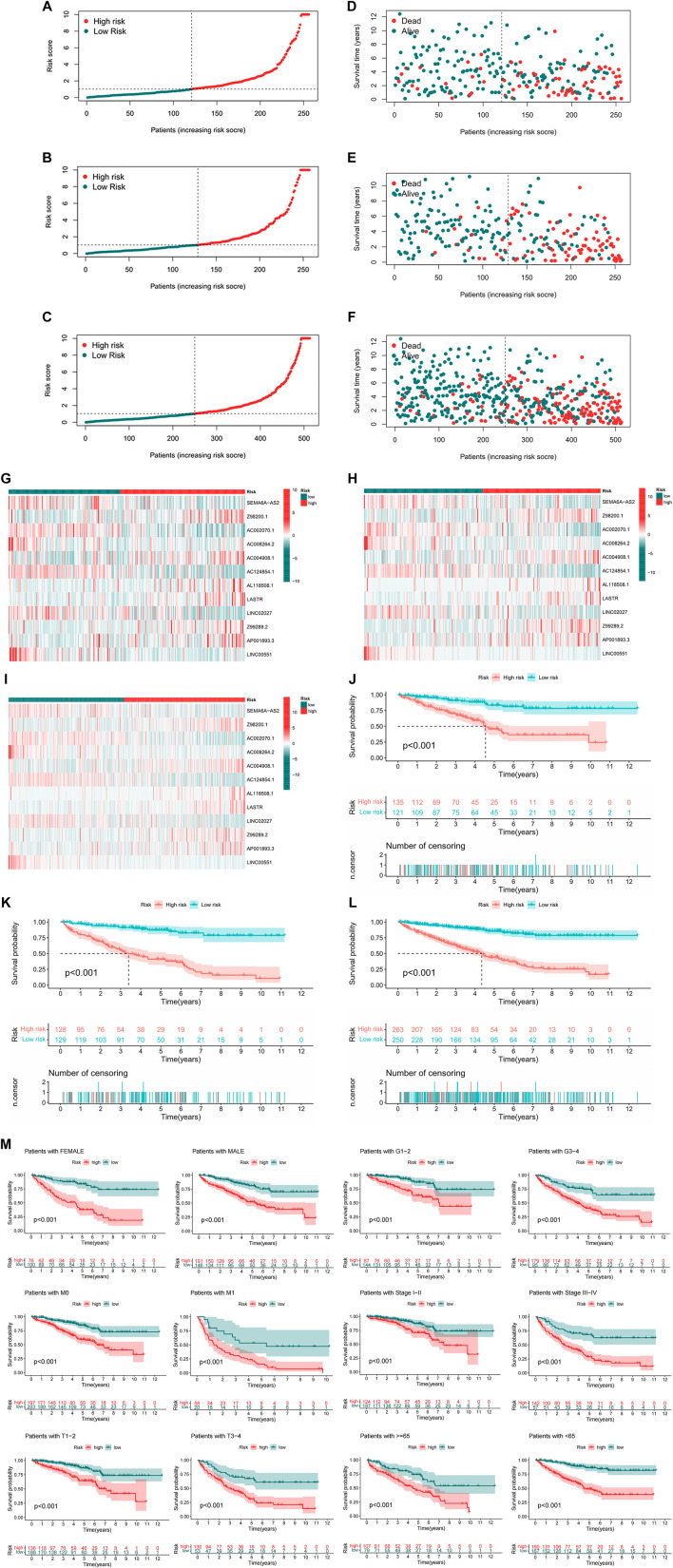
Prognosis value of the 12 m7G-related lncRNAs model in the test, train, and entire set **(A–C)** Exhibition of m7G-related lncRNAs model based on risk score of the test, train, and entire set, respectively, **(D–F)** Survival status and time of patients between two groups in the test, train, and entire set, respectively, **(G–I)** The heatmap of 12 m7G-realted lncRNAs between two groups in the test, train and entire set, respectively, **(J–L)** The survival curve of patients between two groups in the test, train and entire set, respectively, **(M)** Survival curves stratified by age, gender, grade, stage, T, N, or M between two groups in the entire set.

There were specific clinical characteristics of the train set and test set shown in Supplementary Material 6. And then the expression standard of 12 lncRNAs, survival status, survival time were compared between two groups (Figures 4A–L). And it is clear that patients in the high-risk group had worse prognosis than in the low-risk group. Similarly, it also performed the same prognostic results after separated by the clinical parameters (Figure 4M).

### Nomogram

**FIGURE 5:**
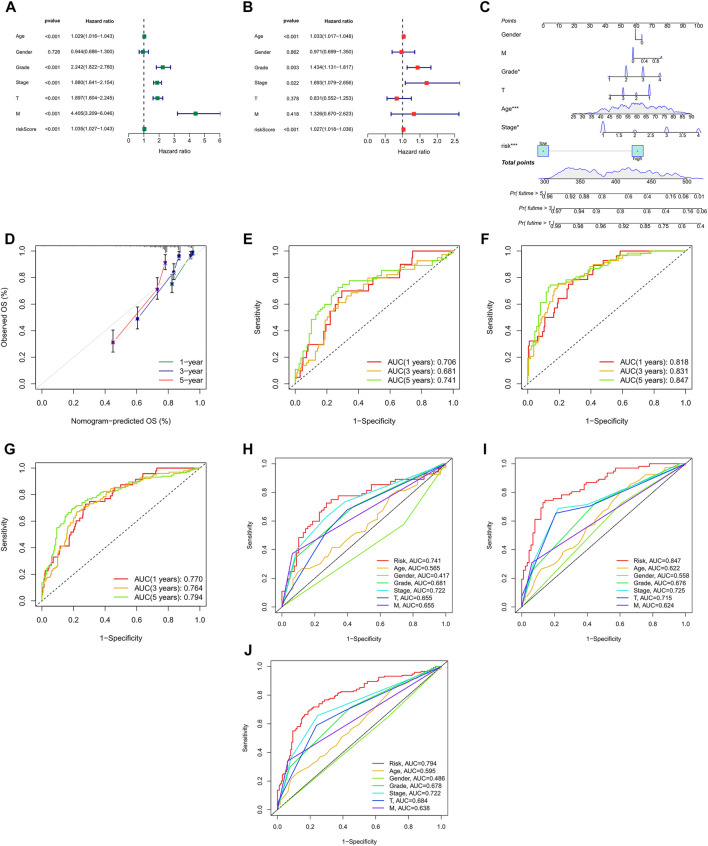
Nomogram and assessment of the risk model **(A,B)** Uni- Cox and multi-Cox analyses of clinical factors and risk score with OS. **(C)** The nomogram that integrated the risk score and clinical parameters to predict the 1-, 3-, and 5-years OS rate. **(D)** The calibration curves for 1-, 3-, and 5-years OS **(E–G)** The ROC curves for 1-, 3-, and 5-years OS rate of the test, train, and entire set, respectively, **(H–J)** The ROC curves for 5-years OS rate of risk score and clinical parameters.

We can see from the univariate Cox regression analysis that age (*HR* = 1.029, *p <* 0.001), tumor grade (*HR =* 2.242, *p <* 0.001), stage (*HR =* 1.880, *p <* 0.001), T stage (*HR =* 1.897, *p <* 0.001), distant metastasis (*HR =* 4.405, *p <* 4.405) and risk score (*HR =* 1.035, *p <* 0.001) were significantly associated with OS (Figure 5A). In contrast, the multivariate Cox regression analysis showed that age (*HR =* 1.033, *p <* 0.001), grade (*HR =* 1.434, *p =* 0.003), stage (*HR =* 1.693, *p =* 0.022) and risk score (*HR =* 1.027, *p <* 0.001) were independent risk factors for OS (Figure 5B).

Incorporating all parameters, we built a nomogram for predicting the 1, 3, 5-years OS rate of all ccRCC patients (Figure 5C). Furthermore, the calibration plots were also used to see if the nomogram was well concordant with the prediction for 1, 3, and 5 years (Figure 5D).

### Assessment of the Model

ROC curves were utilized to evaluate the accuracy of the risk model. It showed that the 1-, 3-, and 5-years area under the ROC curve of the entire set were 0.770, 0.764, and 0.794, of the test set 0.706, 0.681, and 0.741, and of the train set 0.818, 0.831, and 0.847, respectively, (Figures 5E–G). At the 5-years ROC of risk model, it was observed that risk score had a better predictive ability compare with other clinical factors in the entire (0.794), test (0.741) and train set (0.847), respectively, (Figures 5H–J).

### Functional Enrichment Analysis

We divided all of patients into two risk groups according to the risk signature above, and screened 430 differentially expressed m7G-related genes with the criteria of |log_2_ FC| > 1 and *p* < 0.05. Functional enrichment analysis offered a biological understanding of these genes.

**FIGURE 6:**
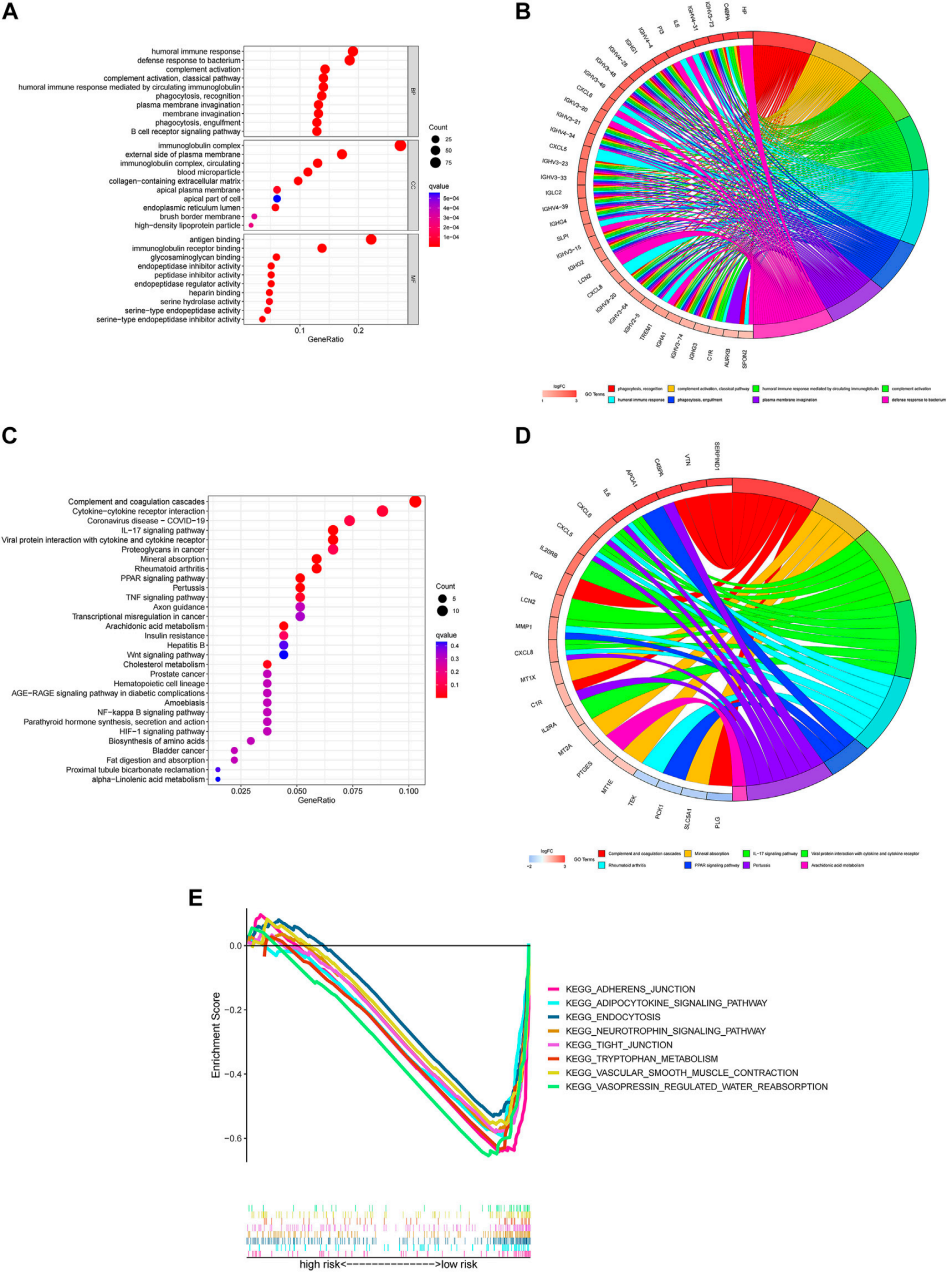
Functional enrichment for differentially expressed m7G genes between two groups **(A)** The top 30 significant terms of GO functional enrichment. BP biological process, CC cellular component, MF molecular function. **(B)** The circle diagram enriched in the GO analysis. **(C)** The top 30 significant terms of KEGG functional enrichment. **(D)** The circle diagram enriched in the KEGG analysis. **(E)** GSEA of the top 8 pathways significantly enriched in the low-risk group.

The GO terms revealed that these genes were significantly associated with humoral immune response, immunoglobulin complex and antigen binding in the in biological processes, cellular components and molecular functions, respectively (Figures 6A,B).

In the KEGG pathway enrichment analysis, these genes were shown to be notably associated with complement and coagulation cascades, cytokine-cytokine receptor interaction and coronavirus disease COVID-19 (Figures 6C,D).

To investigate the biological functions between risk groups, we utilized GSEA software to explore the biological pathways enriched in the high-risk and low-risk group. With the criteria of *p* < 0.05 and FDR <0.25, we got 63 pathways enriched in the low-risk group while there were no pathways enriched in the high-risk group (Supplementary Material 7). Here are the top eight enrichment pathways including adherens junction, adipocytokine signaling pathway, endocytosis, neurotrophin signaling pathway, tight junction, tryptophan metabolism, vascular smooth muscle contraction and vasopressin regulated water reabsorption in the low-risk group (Figure 6E).

### Immunological Analysis

Intriguingly, GO enrichment pathways were significantly associated with immunological functions. Therefore, we further investigated the immunological analysis between two groups.

**FIGURE 7:**
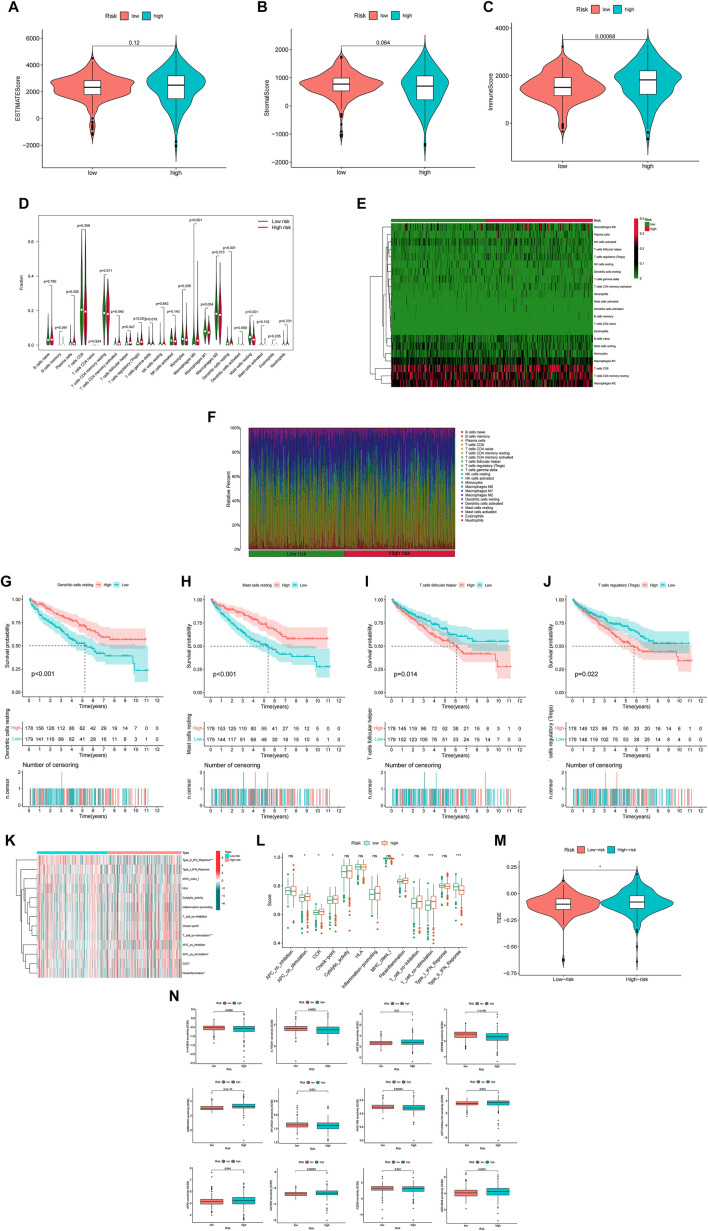
The investigation of tumor immune factors and immunotherapy **(A,B)** The comparison of ESTIMATE scores and stromal scores between two groups. **(C)** The comparison of immune scores between two groups. **(D)** The violin plot for different types of immune cells between two groups. **(E)** The heatmap of immune cells in two groups. **(F)** The bar plot for different types of immune cells in each sample **(G–J)** The survival curves stratified by dendritic cells, mast cells, T cells follicular helper and T cells regulatory (Tregs) **(K)** The heatmap of different immune functions **(L)** The comparison of immune functions between two groups **(M)** The comparison of TIE between two groups **(N)** The immunotherapy prediction of risk groups. Note: ns = non sense. “*” represents *p* < 0.05; “*” *p* < 0.01; “***” *p* < 0.001.

The analysis of TME included ESTIMATE scores and ICL in our study (ESTIMATE scores = stromal component scores + immune cell scores) and the specific immune scores for each patient was in Supplementary Material 8. Although there were no significant differences for ESTIMATE scores and stromal scores between the two risk groups (Figures 7A,B), the high-risk group had higher immune scores than in the low-risk group, which signified a different microenvironment from that in the low-risk group (Figure 7C). Different types of immune cells were associated with the high-risk group on the CIBERSORT platform like plasma cells, T cells CD4 memory activated, T cells regulatory helper, T cells regulatory (Tregs), T cells gamma delta, Macrophages M0, Macrophages M1, dendritic cells resting and Mast cell resting (Figures 7D–F). And we also found dendritic cells resting, mast cells resting, T cells follicular helper and T cells regulatory (Tregs) were significantly associated with OS (Figures 7G–J). Besides, there was also the difference on immune functions between the two risk groups, and it was upregulated for ACP co stimulation, CCR, checkpoint, parainflammation and T cell co-stimulation in the high-risk group while type II IFN response downregulated (Figures 7K,L). In addition, patients in the high-risk group were prone to have higher TIE scores compared those in the low-risk group (Figure 7M). What’s more, the high-risk patients had a lower IC50 of 12 immunotherapeutic drugs such as A-443654, A-770041, ABT-263 and so on (Figure 7N).

### Tumor Mutation Burden

**FIGURE 8:**
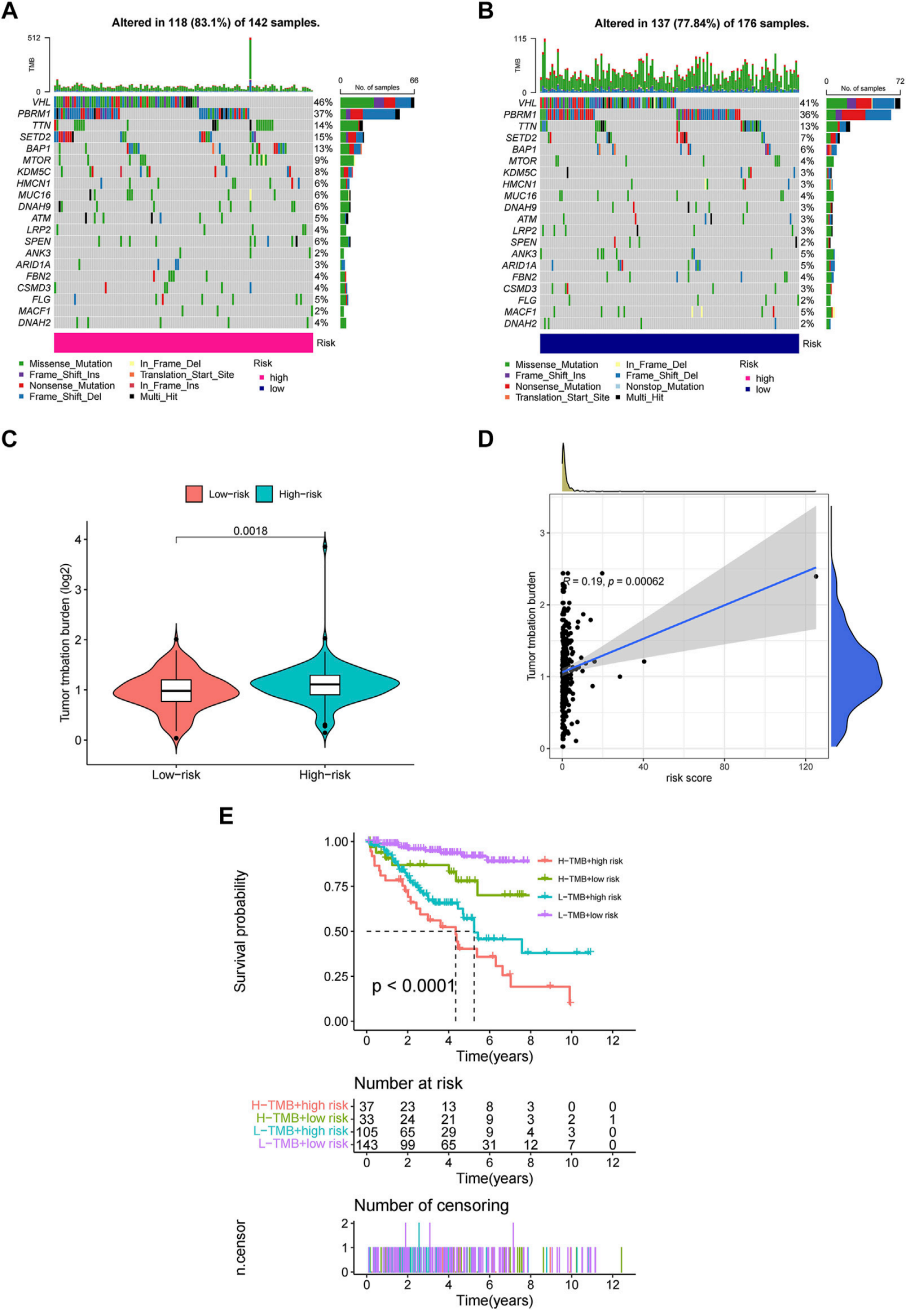
The investigation of tumor mutation burden (TMB) **(A,B)** TMB in the high-risk and low-risk group, respectively. **(C)** The comparison of TMB between two groups **(D)** Correlation between TMB and risk scores **(E)** The survival curve stratified by TMB and risk signature.

Employing the data of tumor mutation from TCGA, we got the mutation rate for each gene and TMB for each sample (Supplementary Material 1). It can be seen that regardless of the subgroup, VHL has the highest mutation rate of more than 40% in ccRCC, followed by PBRM1, TTN, SETD2, etc. (Figures 8A,B). It also showed that TMB in the high-risk group was significantly higher than in the low-risk group (Figure 8C). Moreover, TMB was also positively correlated with risk cores (Figure 8D), and higher TMB indicated worse prognosis for ccRCC patients (Figure 8E).

## Discussion

Renal cell carcinoma (RCC) is the seventh most frequently diagnosed cancer in the world. As the dominant pathological subtype, ccRCC has a higher risk of recurrence, metastasis, and worse prognosis. Some prognostic models based on m6A RNA modification-related lncRNAs could improve the understanding and management of ccRCC (Ma et al., 2021a; Ma et al., 2021b). However, there have been no models based on m7G-related lncRNAs for ccRCC. In this study, we screened the target genes in 4 databases (Gene Cards, OMIM, NCBI, GSEA) and developed a reliable prognostic model with satisfactory predictive value for ccRCC.

We downloaded RNA-seq and clinical data from 539 tumor samples and 72 normal tissue samples from TCGA. Using LASSO and Cox regression analysis, we screened a total of 12 m7G-related lnRNAs to establish the risk signature and found that patients in the high-risk group had a worse prognosis. And integrating the clinical parameters and risk scores, we constructed the nomogram for predicting the prognosis. Next, functional enrichment analysis was conducted. In KEGG enrichment analysis, complement and coagulation cascades ranked in the top, which played an important role in the progression of malignant tumors, such as breast cancer, liver cancer, etc. (Guglietta and Rescigno, 2016; Zhang et al., 2020; Zhang et al., 2021) Similarly, some of the pathways enriched in the low-risk group in GSEA were also malignancy-related pathways such as adherens junction (Bischoff et al., 2020; Wei et al., 2020), endocytosis (Polo et al., 2004; Khan and Steeg, 2021), tight junction (Runkle and Mu, 2013; Kyuno et al., 2021) and so on. Notably, there were many of enrichment pathways related immune activities like humoral immune response, defense response to bacteria, immunoglobulin complex and antigen binding. Therefore, we further conducted the immunological analysis to investigate the relationship between m7G-related lncRNAs and immunology.

In recent years, the emerging role of lncRNAs has drawn increasing attention in tumor initiation and progression. Among the 12 m7G-related lncRNAs we screened, 3 lncRNAs (AC124854.1, LASTR and LINC00551) exhibited pro-tumorigenic effects. Gong et al. developed a prognostic model for predicting ccRCC prognosis based on 10 lncRNAs, of which the prognostic value of AC124854.1 has been confirmed in this study (Cheng et al., 2019). LASTR, upregulated in hypoxic breast cancer, contributes to triple-negative breast cancer cell fitness by regulating the activity of the U4/U6 recycling factor SART3 (De Troyer et al., 2020). The expression of lINC00551 inhibited development of esophageal squamous cancer through reducing the level of HSP27 phosphorylation (Peng et al., 2021), and suppressed lung adenocarcinoma progression by regulating c-Myc-mediated PKM2 expression (Wang et al., 2020). However, it also showed tumor-promoting effects of lINC00551 in non-small cell lung cancer (Zhu et al., 2019). For the remaining 9 lncRNAs, no relevant reports in tumors have been found, which might provide us with new research directions to consult the potential roles in cancer in the future.

Due to its complexity and continuous evolution in tumorigenesis and progression, it has gained much attention for TME comprising stromal cells, fibroblasts, and endothelial cells, innate immune cells (macrophages, neutrophils, dendritic cells, innate lymphoid cells, myeloid-derived suppressor cells, and NK cells) and adaptive immune cells (T cells and B cells) in recent years (Hinshaw and Shevde, 2019). In our study, tumors with higher ESTIMATE scores in the high-risk group suggested a more complex TME. It could be observed that 5 types of immune cells (plasma cells, T cells CD4 memory activated, T cells regulatory helper, T cells regulatory and Macrophages) were upregulated in the high-risk group while 4 types of them (T cells gamma delta, Macrophages M1, dendritic cells resting and Mast cell resting) downregulated in our study. In most solid tumors, tumor-associated macrophages (TAMs) formed the majority of the myeloid infiltrate, and could be polarized into M1 and M2 subset when exposed to different cytokines. M1 subset was involved in the inflammatory response, clearing pathogens from the body and participating in anti-tumor immunity, while M2 subset functioned in the anti-inflammatory response, repaired tissue damage and promoted tumor progression (Hinshaw and Shevde, 2019; Vitale et al., 2019). Dendritic cells (DC) were able to recognize, capture and present antigens to activate CD4^+^ and CD8^+^ T cells, which built a bridge between innate and adaptive immunity (Fu and Jiang, 2018). Probst et al. found that resting dendritic cells could induced peripheral CD8^+^ T cell tolerance through PD-1 and CTLA-4 in mice (Probst et al., 2005). B and plasma cells may induce anti-tumor effects by triggering the complement cascade and antibody-dependent cell cytotoxicity (ADCC) (Kinker et al., 2021). In the tumor microenvironment, T cells had complex functions due to their different subtypes. Regulatory T cells (Tregs) could attenuate effector T cell activity and promote immunosuppression (Najafi and Mirshafiey, 2019; Huppert et al., 2022). CD8^+^ T cells have been considered as a positive prognostic biological maker because of its cytotoxic and tumor-killing capabilities. Immune checkpoints were the overexpressed inhibitory receptors on CD8^+^ T cells such as PD-1, CTLA-4, TIM-3, Lag-3 and TIGHT, which could drive CD8^+^ T cell dysfunction and thus promote immune escape of tumors (Joyce and Fearon, 2015; Durgeau et al., 2018; Thommen and Schumacher, 2018). At present, ICB significantly improved prognosis of patients for various types of cancer, like Ipilimumab for advanced melanoma, Nivolumab for non-small-cell lung cancer, Atezolizumab for urothelial cancer, Cabozantinib for ccRCC and others. It has been found that ICB could regulate the abundance and function of CD4^+^ T cell which reduced the number of Tregs and synergized the anti-tumor effects of CD8^+^ T cells (Li et al., 2020). γδ T cells were able to inhibit the recruitment and activation of αβ T cells, thus promoting oncogenesis and progression of pancreatic ductal adenocarcinoma (PDA) (Daley et al., 2016; Hundeyin et al., 2019). Therefore, immunotherapy for the remodeling of the tumor microenvironment has been regarded as a promising but challenging approach to cancer treatment.

TMB refers to the overall number of somatic mutations that occur in a defined area of a tumor genome (Alexandrov et al., 2013; Schumacher and Schreiber, 2015; Chan et al., 2019). Since early studies were mostly based on whole exome sequencing (WES) which was also the gold standard for TMB detection, TMB usually was usually reported as the total number of coding and somatic mutations. Afterwards, TMB detection has developed from WES to the more clinically relevant targeted next-generation sequencing panel (NGS panel) where it was also defined as the sum of base substitution, insertion and deletion mutations per megabase (mut/Mb) in the coding region of targeted genes (Chan et al., 2019). Some somatic mutations could be transcribed and translated, which means higher TMB could indirectly reflect the ability of tumors to form more neoantigens (Schumacher and Schreiber, 2015; Riaz et al., 2016). Although not all neoantigens were immunogenic, these neoantigens could be recognized and targeted by immune system, and then induce T-cell cytotoxicity and anti-tumor response, which in turn increased the sensitivity of ICB. Some studies have reported TME could be used as a biomarker for the therapeutic effectiveness of ICB (Gandara et al., 2018; Wang et al., 2019; Marabelle et al., 2020).

We used clinical and survival data from a large number of ccRCC patients in TCGA database to develop a prognostic model of m7G-related lncRNAs and validated it internally. However, there are also some limitations for this study. First, it is a retrospective analysis and there is avoidable selection bias and recall bias. Second, although employing other databases such as CIBERSORT for immunological analysis, we did not combine the cohort from other databases for further external validation. Thus, we still need larger sample size and prospective clinical trial for further validation of the model in the future.

In conclusion, our study identified 12 m7G-related lncRNAs with prognostic value, developed a prognostic and predictive m7G-related lncRNA prognostic signature, which is likely to be useful in elucidating the functional and molecular mechanisms in oncogenesis and progression of ccRCC, as well as determining the appropriate and optimal treatment for patients.

## Data Availability

The datasets presented in this study can be found in online repositories. The names of the repository/repositories and accession number(s) can be found in the article/Supplementary Material.
